# Extraarticular distal humeral nonunion: systematic review of literature

**DOI:** 10.1186/s10195-025-00861-y

**Published:** 2025-07-21

**Authors:** Giovanni Vicenti, Enrico Guerra, Elisa Pesare, Giulia Colasuonno, Marco Minerba, Michele Loiodice, Francesco Conte, Paolo Sergi, Giuseppe Solarino

**Affiliations:** 1 Orthopedic and Trauma Unit, Department of Translational Biomedicine and Neuroscience (DiBraiN), School of Medicine, University of Bari Aldo Moro, AOU Consorziale “Policlinico”, 70124 Bari, Italy; 2 Shoulder and Elbow Unit, IRCCS Istituto Ortopedico Rizzoli, 40100 Bologna, Italy

**Keywords:** Distal humeral nonunion, Open reduction and internal fixation (ORIF), Total elbow arthroplasty (TEA), Healing rate, Infection management, Revision surgery, Surgical complications

## Abstract

**Background:**

Distal humeral fractures accounted for ~1% of all fractures; however, they were prone to complications, including nonunion if left untreated or inadequately managed. Nonunion, which predominantly occurred at the supracondylar level, resulted in mechanical instability, functional impairment, and persistent discomfort. The most commonly employed surgical options included open reduction and internal fixation (ORIF), total elbow arthroplasty (TEA), and external fixation. This article provides a comprehensive assessment of these surgical procedures and shared clinical experiences related to these challenging cases.

**Methods:**

A systematic review of literature was conducted using the PubMed database up to October 2024, with a focus on cases involving extraarticular distal humeral nonunions that were treated with ORIF, TEA, or Ilizarov techniques.

**Results:**

A total of 25 studies involving 448 patients were encompassed in the review, with a mean patient age of 50 years and an average follow-up period of 48 months. Reported success rates for ORIF and TEA were 90% and 74%, respectively. A higher rate of fracture healing was demonstrated by ORIF, although functional outcomes were found to be comparable between the techniques. Complications such as infections and reduced range of motion (ROM) were documented.

**Conclusions:**

The highest success rate in treating aseptic nonunions was associated with ORIF, highlighting the importance of stable fixation, bone grafting, and meticulous preoperative planning. TEA was regarded as a viable option, particularly for patients with poor bone quality or complex, unreconstructible fractures. To optimize outcomes, surgical techniques were required to be customized on the basis of patient-specific factors and surgeon expertise. Further research is recommended to facilitate the comparison of long-term functional outcomes across different surgical approaches.

*Level of evidence*: IV.

**Supplementary Information:**

The online version contains supplementary material available at 10.1186/s10195-025-00861-y.

## Introduction

Distal humeral fractures account for ~1% of all fractures, exhibiting a bimodal distribution with peaks in pediatric and older populations (over 60 years). Surgical intervention is the preferred treatment method for these fractures. Nonoperative management tends to be ineffective, increasing the risk of nonunion sixfold and leading to suboptimal functional outcome[1].

Nonunions of the distal humerus typically occur at the supracondylar level, where the articular fragments may heal in near-anatomical alignment. Successful treatment of supracondylar fractures depends on achieving adequate compression between the diaphysis and the articular segment during surgical reduction and fixation. This region experiences significant torsional and deforming forces during everyday activities and therefore requires sufficient stability and mechanical stiffness for effective healing. Inadequate support in the supracondylar area correlates with increased rates of failure and nonunion [2–5].

Reported nonunion rates for distal humeral fractures range from 2% to 10% [6, 7]. Nonunions can be classified as septic or aseptic, with various patient and fracture-specific factors influencing the risk of complications. Inadequate fixation, complex fracture patterns, poor bone quality, soft tissue injuries, and patient-related risk factor selection are all recognized predictors of nonunion [7–11]. Motion at the nonunion site can lead to discomfort, reduced elbow function, and increased disability over time. The presence of screws may also lead to loosening or failure, further compromising bone integrity.

Oligotrophic nonunions of the distal humerus are characterized by a combination of reduced biological activity and insufficient stability. It is also essential to check for infection even in the absence of overt clinical signs such as fever, discharge, or wound complications [12, 13].

Management options for distal humeral nonunions include nonoperative management, open reduction and internal fixation (ORIF), and total elbow arthroplasty (TEA). The choice of management should be tailored to the patient’s needs and quality of life considerations [14]. Conservative treatment may be appropriate for low-demand individuals who are unable to undergo surgery owing to significant comorbidities; however, this approach is associated with higher rates of persistent nonunion and infection. By contrast, ORIF is generally recommended for the majority of distal humeral nonunions [15]. ORIF can also serve as a secondary intervention that incorporates auto-allograft in cases of bone loss after the treatment of septic nonunion, as suggested by Brinker et al. [16].

For patients with unreconstructable joints, arthrodesis may be considered as a salvage procedure to provide stability and pain relief, especially in cases of ongoing deep infection or compliance issues that preclude arthroplasty [17]. While TEA offers a less invasive surgical option with more straightforward postoperative rehabilitation, it is typically reserved for specific patient populations, as mechanical failures in younger patients can lead to significant complications. Therefore, the aim of this article is to conduct a systematic review of the current literature on surgical treatments for distal humeral nonunions, comparing the functional outcomes of various treatment strategies, such as ORIF and TEA, with a specific focus on patient characteristics and needs.

## Methods

This systematic review was conducted in accordance with the Preferred Reporting Items for Systematic Reviews and Meta-analyses (PRISMA) guidelines[18]. The study protocol was registered in PROSPERO (CDR656075). Searches were performed in the PubMed and CENTRAL databases, covering the period from January 1982 to October 2024. The research question was formulated using the PICO framework:

- P: patients with nonunion extraarticular distal humerus.
- I: ORIF; bone grafting; Ilizarov techniques; TEA.
- C: no specific control.
- O: healing at 2 years follow-up; range of movement (including complication).

The following search string was used: ((“Humeral Fractures”[MeSH] OR “Fractures, Ununited”[MeSH] OR “humerus fracture nonunion”[tiab] OR “distal humerus nonunion”[tiab] OR “pseudoarthrosis of the humerus”[tiab]) AND (“Fracture Fixation, Internal”[MeSH] OR “Open Reduction Internal Fixation”[tiab] OR “ORIF”[tiab] OR “Bone Transplantation”[MeSH] OR “bone graft”[tiab] OR “Ilizarov Technique”[MeSH] OR “Ilizarov”[tiab] OR “Arthroplasty, Replacement, Elbow”[MeSH] OR “Total Elbow Arthroplasty”[tiab] OR “TEA”[tiab]) AND (“Fracture Healing”[MeSH] OR “Treatment Outcome”[MeSH] OR “Range of Motion, Articular”[MeSH] OR “functional outcome”[tiab] OR “range of motion”[tiab] OR “complications”[tiab])). Terms searched were “distal humerus fractures,” “nonunion,” “treatment,” “ORIF,” and “TEA”.

### Study selection and eligibility criteria

The literature search strategy employed to identify relevant studies was conducted following the Medical Subject Headings (MeSH) (strategy, and details of this approach are available as supplementary material). Eligible study designs included randomized controlled trials (RCTs), cohort studies (both prospective and retrospective), and case reports. Studies involving patients treated for distal humerus nonunions were included. Only full-text articles published in English were considered. Editorials, commentaries, surveys, animal-only studies, in vitro experiments, cadaveric analyses, biomechanical investigations, studies focusing on humeral posttraumatic osseous defects, and research involving pediatric populations were excluded. Narrative reviews, systematic reviews, letters to the editor, and expert opinions were also excluded. Articles that lacked sufficient information on postoperative outcomes were omitted. No filters or additional constraints were applied during the database search. The secondary outcome assessed was range of motion (ROM) and functionality.

### Data extraction

Two reviewers (P.E. and G.C.) independently evaluated the retrieved citations on the basis of predetermined inclusion and exclusion criteria. Disagreements were settled by discussion or by a third party (G.V. and E.G.) with great expertise on the topic. The following data were collected from the included papers: study characteristics (study design, duration, year of publication, and sample size), participants’ characteristics (age and sex), surgical approach and technique, treatment, success rate (%), and complications.

The level of evidence was assessed using the Oxford Center for Evidence-Based Medicine (OCEBM) 2011 Levels of Evidence [19]. Risk of bias was assessed independently by two reviewers (P.E. and G.C.) using the Risk of Bias in Nonrandomized Studies of Intervention (ROBINS-I) tool for observational studies, and the Joanna Briggs Institute Checklist for the two case reports were applied. With the aid of these tools, various forms of bias were evaluated, including confounding bias, selection bias, bias in classification of intervention, bias due to deviations from intended interventions, bias due to missing data, bias in measurement of outcome, and bias in selection of the reported results.

ROBINS-I for nonrandomized controlled trial (nRCT)/Joanna Briggs Institute Critical Appraisal Checklist for case reports are presented in Table 1 and in Table 2. Characteristics of the included research papers (i.e., first author, publication year, study design, and type of nonunion studied) are presented in Table 3. Main outcomes of included studies are presented in Table 4.

**Table 1:** Risk of bias in nonrandomized studies of intervention (ROBINS-I) tool for non-RCT

Refs.	Confounding	Classification of intervention	Selection of participants	Deviation from intended interventions	Missing data	Measurements of outcomes	Selections of reported results	Overall
Ackerman et al. [25]	NI	Moderate	Moderate	Low	Moderate	Low	Low	Moderate
Sanders et al. [26]	NI	Low	Moderate	Low	Moderate	Moderate	Low	Moderate
McKee et al. [27]	NI	Low	Moderate	Low	Low	Moderate	Low	Moderate
Simonis et al. [28]	NI	Moderate	Moderate	Low	Moderate	Moderate	Low	Moderate
Helfet et al. [29]	NI	Low	Moderate	Low	Low	Low	Moderate	Moderate
Ali et al. [30]	NI	Low	Moderate	Low	Moderate	Low	Low	Moderate
Allende et al. [8]	NI	Low	Moderate	Low	Moderate	Moderate	Low	Moderate
Niu et al. [31]	NI	Moderate	Low	Moderate	Moderate	Low	Low	Moderate
Ouyang et al. [32]	NI	Moderate	Moderate	Low	Low	Low	Moderate	Moderate
Figgie et al. [20]	NI	Low	Moderate	Low	Moderate	Moderate	Low	Moderate
Cil et al. [21]	NI	Moderate	Low	Low	Moderate	Low	Low	Moderate
LaPorte et al. [22]	NI	Low	Moderate	Low	Moderate	Moderate	Low	Moderate
Pogliacomi et al. [23]	NI	Low	Low	Low	Moderate	Low	Moderate	Moderate
Morrey et al. [33]	NI	Low	Moderate	Low	Moderate	Moderate	Low	Moderate
Brinker et al. [16]	NI	Low	Moderate	Low	Low	Low	Low	Moderate
Cavadas et al. [34]	NI	Low	Low	Moderate	Moderate	Moderate	Low	Moderate
Mitsunaga et al. [24]	NI	Low	Moderate	Low	Moderate	Moderate	Low	Moderate
Jupiter and Goodman et al. [35]	NI	Low	Moderate	Low	Moderate	Moderate	Low	Moderate
Kerfant et al. [36]	NI	Low	Moderate	Low	Moderate	Moderate	Low	Moderate
Tomic et al. [39]	NI	Low	Moderate	Low	Moderate	Moderate	Moderate	Moderate
Leiblein et al. [41]	NI	Moderate	Moderate	Low	Moderate	Low	Low	Moderate
Abhiram et al. [7]	NI	Low	Moderate	Low	Moderate	Moderate	Low	Moderate
Pullen et al. [38]	NI	Moderate	Moderate	Low	Low	Moderate	Low	Moderate

*NI* no information

**Table 2:** Joanna Briggs Institute critical appraisal checklist for case reports (*n* = 2)

JBI checklist questions	Zafra et al. [37]	Bigoni et al. [40]
1. Were the patient’s demographic characteristics clearly described?	Yes	Yes
2. Was the patient’s history clearly described and presented as a timeline?	No	No
3. Was the current clinical condition of the patient on presentation clearly described?	Yes	Yes
4. Were diagnostic tests or assessment methods and the results clearly described?	Yes	Yes
5. Was the intervention(s) or treatment procedure(s) clearly described?	Yes	Yes
6. Was the postintervention clinical condition clearly described?	Not applicable	Not applicable
7. Were adverse events (harms) or unanticipated events identified and described?	Yes	Yes
8. Does the case report provide takeaway lessons?	Yes	Yes
Overall appraisal	Included	Included

Scoring: yes, no, unclear, or not applicable. *JBI* Joanna Briggs Institute

**Table 3:** Characteristics of included studies

Ref	Year of publication	Infected/noninfected nonunion	Type of study (prospective/retrospective study)	*N* (patients)	Male/Female	Mean age (years)	Mean follow-up (months)	Oxford level of evidence
Ackerman et al. [25]	1998	Noninfected nonunion	Retrospective	20	–	–	36	Level IV
Sanders et al. [26]	1990	Noninfected nonunion	Retrospective	7	2/3	52	–	Level IV
McKee et al. [27]	1994	Noninfected nonunion	Retrospective	13	6/7	39	25	Level IV
Simonis et al. [28]	2003	Noninfected nonunion	Retrospective	14	5/9	55	10	Level IV
Helfet et al. [29]	2003	Noninfected nonunion	Retrospective	52	–	47	33	Level IV
Ali et al. [30]	2005	Noninfected nonunion	Retrospective	16	10/6	47	39	Level III
Allende et al.[8]	2009	Noninfected nonunion	Retrospective	24	16/8	45	46	Level IV
Niu et al. [31]	2012	Noninfected nonunion	Retrospective	22	14/8	34	39	Level IV
Ouyang et al. [32]	2013	Noninfected nonunion	Retrospective	11	–	41	5.6	Level IV
Figgie et al. [20]	1989	Noninfected nonunion	Retrospective	14	–	42	24	Level IV
Cil et al. [21]	2008	Noninfected nonunion	Retrospective	91	1/5	68	18	Level IV
LaPorte et al. [22]	2008	Noninfected nonunion	Retrospective	5	–	60	14	Level IV
Pogliacomi et al. [23]	2015	Noninfected nonunion	Retrospective	20	1/0	39	20	Level IV
Morrey et al. [33]	1995	Noninfected nonunion	Retrospective	26	12/14	59	60	Level IV
Brinker et al.[16]	2007	Noninfected nonunion	Prospective	7	4/3	53	22	Level IV
Cavadas et al. [34]	2010	Noninfected nonunion	Retrospective	5	2/2	32	47	Level IV
Mitsunaga et al. [24]	1982	Noninfected nonunion	Retrospective	32	1/0	58	24	Level IV
Jupiter and Goodman et al. [35]	1992	Noninfected nonunion	Retrospective	6	–	68	71	Level IV
Kerfant et al.[36]	2012	Noninfected nonunion	Retrospective	5	2/3	50	48	Level V
Zafra et al. [37]	2015	Noninfected nonunion	Retrospective	1	1/0	65	60	Level IV
Bigoni et al. [40]	2019	Infected nonunion	Retrospective	1	1/0	65	72	Level IV
Tomic et al. [39]	2017	Infected nonunion	Retrospective	19	11/8	61	63	Level IV
Leiblein et al. [41]	2019	Both noninfected and infected nonunion	Retrospective	26	14/12	55	72	Level IV
Abhiram et al.[7]	2019	Noninfected nonunion	Retrospective	7	–	57	67	Level IV
Pullen et al.[38]	2003	Infected nonunion	Retrospective	4	–	56	24	Level IV

**Table 4:** Main outcomes of included studies

Refs.	Surgical treatment	Procedure and approach	Patient (*N*)	Success rate % (*N*)	Complications	ROM functionality outcome
Ackerman et al. [25]	ORIF	Posterior	20	94% (18)	Two excisions of distal humerus and replacement with allograft	1 Excellent 6 Good 7 Fair 6 Poor
Simonis et al. [28]	ORIF	Posterior	14	85% (12)	Two required removal of metalwork	2 Excellent 7 Good 2 Fair 2 Poor
Ali et al. [30]	ORIF	Posterior	16	99% (15)	One infection	11 Excellent 2 Good 2 Fair 1 Poor
Cavadas et al. [34]	ORIF and vascularized bone transfer	Posterior	5	96% (4)	One septic complication	1 Excellent 3 Good 1 Fair 1 Poor
Sanders et al. [26]	ORIF and bone graft	Posterior	7	71% (5)	2 Necrosis trochlea	2 Good 2 Fair 1 Poor
McKee et al. [27]	ORIF and bone graft	Posterior	7	100% (7)	–	1 Excellent 4 Good 2 Fair
Helfet et al. [29]	ORIF and bone graft	Posterior	52	99% (51)	Two superficial infections Two deep infections Five ulnar neuropathies	51 Good
Jupiter and Goodman et al. [35]	ORIF and bone graft	Posterior	6	83% (5)	One case of avascular necrosis of the trochlea	5 Good 1 Fair
Kerfant et al. [36]	ORIF with vascularized bone graft	Lateral approach (Gilbert)	5	100% (5)	No complications	–
Zafra et al. [37]	ORIF with vascularized bone graft	Posterior	1	100% (1)	–	–
Niu et al. [31]	ORIF an bone graft	Posterior	22	100% (22)	Two postoperative heterotopic calcification One superficial infection Two ulnar nerve injuries	8 Excellent 9 Good 4 Fair 1 Poor
Abhiram et al. [7]	ORIF and autogenous grafting	–	7	100% (7)	–	5 Excellent 1 Good 1 Poor
Allende et al. [8]	ORIF/ ORIF and graft (15 patients)	Posterior in 21 (trans olecranon in 11, posterior triceps elevating approach (Bryan–Morrey) in 4, posterior triceps-on (Alonso-Llames) in 6); lateral humerus approach in 2, a Kocher lateral approach in 1	24	100% (24)	Six infections treated with a two-stage reconstruction: cement spacer with antibiotics, ORIF, and bone graft One olecranon fracture Three transpositions of the ulnar nerve Six removals of metalwork	23 Good 1 Poor
Ouyang et al. [32]	ORIF and hinged external fixation and bone graft	Posterior and external fixation unilateral access	11	100% (11)	–	6 Excellent 3 Good 2 Fair
Morrey et al. [33]	22 ORIF 7 TEA	–	39	86% (31)	Seven complications with five reoperations	31 Good 3 Fair 2 Poor
Mitsunaga et al. [24]	25 ORIF 7 TEA	Lateral “J” approach of Kocher in 11, posterior in 3, triceps splitting in 9, medial-to-lateral reflection in 9	32	56% (ORIF 14) 71% (TEA 5)	Six revisions with bone graft (ORIF cases) Two revisions for loosening (TEA)	–
Figgie et al. [20]	TEA	Posterior	14	57% (8)	Three revisions for infection/mobilization	–
Cil et al. [21]	TEA	Posterior in 16, subperiosteally reflected in 20, partially reflected in 4, and split in 3	91	74% (67)	44 complications (23 reoperations)	77 Good 20 Fair
LaPorte et al. [22]	TEA	Standard posterior/Bryan–Morrey triceps-sparing	12	75% (11)	Two reoperations	4 Excellent 6 Good 1 Poor
Pogliacomi et al. [23]	TEA	Splitting triceps/Bryan–Morrey triceps-sparing	20	80% (16)	Si complications One superficial wound infection One deep infection	15 Excellent 4 Good 1 Poor
Leiblein et al. [41]	Both noninfected and infected non union: 2 Ilizarov External Fixator, 3 TEA, 2 nailing and allograft, 17 ORIF and bone graft, 2 no treatment	–	26	61% (16)	Ten lost to follow-up Seven reached Complete consolidation, two reached partial consolidation Three did not show consolidation	–
Bigoni et al. [40]	Two-stage reconstruction: bone and soft tissue debridment and then ORIF with vascularized bone graft	Posterior	1	100% (1)	–	–
Tomic et al. [39]	Ilizarov external fixator	External fixation unilateral access	19	100% (19)	Complication: two nerve paresis, eight postoperative infections	6 Good 13 Fair
Brinker et al. [16]	Ilizarov External Fixator	One-stage: external fixation unilateral access and iliac crest harvesting	6	83% (5)	One refractured	5 Good
Pullen et al. [38]	Ilizarov external fixator	External fixation unilateral access	4	100% (4)	–	–

## Results

**Fig. 1:**
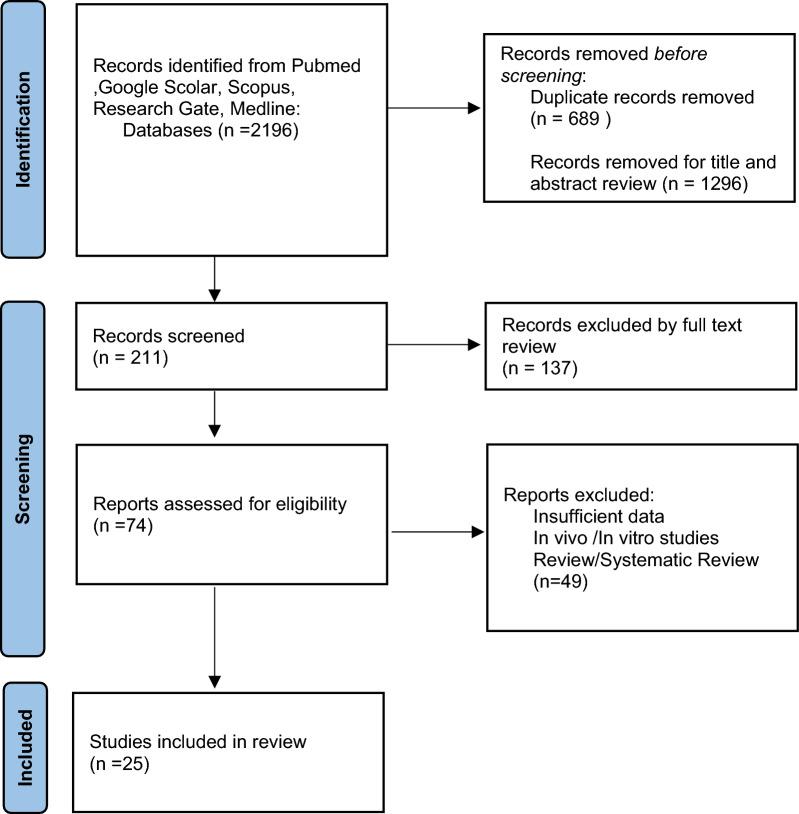
PRISMA flow diagram

The literature search yielded 2196 results. The selection process is reported in detail in the PRISMA flow diagram (Fig. 1—PRISMA Flow diagram). Overall, these 25 studies comprising 448 patients undergoing surgical revision for distal humeral nonunion were analyzed. The average age of the patients included in the study was 50 years, ranging from 32 to 68 years, with a mean follow-up duration of 48 months (Table 4). Surgical procedures for distal humerus nonunion consisted of TEA in six studies [5, 20–24] and ORIF with synthetic or vascularized bone graft in 16 studies [7, 8, 24–37]. Additionally, Ilizarov external fixation was used in 31 infected patients across six articles [16, 38–41].

Patient diagnoses, including both infected and noninfected nonunion types, were detailed in all articles. A total of 4 of the 25 articles [16, 38–40] focused exclusively on infected nonunion, while one [41] addressed both. All studies were retrospective, involving 154 patients, 80% of whom were female, with a mean age of 60.4 years (range: 55–65) and an average follow-up of 60 months (Table 4). A total of two studies involved both TEA and ORIF procedures.

The TEA success rate was 74%, with 107 out of 144 patients healed by the final follow-up. Functional outcomes, measured by the Disabilities of the Arm, Shoulder, and Hand (DASH) and Mayo Elbow Performance Score (MEPS), revealed that about 67% of patients achieved good functionality, while 14% exhibited excellent functionality; 17% demonstrated only fair functionality (Table 4).

In total, 14 studies investigated ORIF [3, 7, 8, 25–29, 31, 32, 34–37]. The cohort consisted of 272 individuals who underwent surgical revision for distal humeral nonunion via ORIF with either vascularized or synthetic grafts; 56% were men. The average age was 51 years (range: 34–68), with a mean follow-up of 25 months (Table 4). Only five papers included patients with infected nonunion. This cohort consisted of 32 individuals, with 54% being male, and an average age of 45 years (range: 32–58), with an average follow-up of 47 months (Table 3).

## Discussion

### Noninfected nonunion

#### Total elbow arthroplasty (TEA)

TEA was considered the treatment of choice for humeral fractures with an unreconstructable distal articular surface, particularly in cases where stable fixation could not be achieved with ORIF owing to small fragment sizes, highly comminuted fractures, or poor bone quality. TEA was not recommended for younger patients or in cases of suspected septic nonunion [21]. Contraindications included neurological damage, impaired hand function, dementia, and noncompliant patients. Relative contraindications encompassed open fractures and weight-bearing considerations in the upper extremity.

Distal humerus hemiarthroplasty was regarded as an attractive option for younger individuals with unreconstructible fractures; however, its utilization for nonunions was reported infrequently. The available data indicated that, although TEA was effective in alleviating pain and restoring a certain degree of function, a substantial proportion of patients (approximately 19%) did not achieve satisfactory functional outcomes. This limitation was particularly relevant for patients with higher activity expectations. Additional caution was required for patients with preexisting arthrosis or inflammatory arthritis.

The studies reviewed were characterized by a mean follow-up duration of approximately 60 months, which enabled a comprehensive assessment of long-term outcomes.

#### Open reduction and internal fixation (ORIF)

A notably high success rate was observed within the ORIF group, reaching 90%, with 252 out of 272 patients achieving union by the final follow-up. In the analysis conducted by Morrey et al., both TEA and ORIF procedures were included; however, no specific data regarding differences in success rates between these two interventions were reported. Additionally, information on differences in outcomes between infected and noninfected patients was not provided by Leblein et al.; consequently, these groups were excluded from the success rate analysis.

Functional outcomes, assessed using the DASH score and MEPS where applicable, indicated that approximately 67% of patients demonstrated good functionality (Table 4).

In cases of nonunion, favorable results were reported with revision ORIF when the joint surface was preserved [30]. Morrey et al. developed a decision-making algorithm aimed at predicting treatment outcomes for nonunion or malunion of the distal humerus, based on several key conditions that needed to be satisfied for performing revision ORIF. Firstly, the articular surface was required to be intact and congruent; if this condition was not met, TEA was recommended instead. The second condition concerned the viability and biological stability of the fracture fragments. The supracondylar region was most frequently involved in the development of nonunion, primarily due to micromotion at the fracture ends and limited bone-to-bone contact, which often led to fixation failure. High stability and compression were required in this region to withstand the significant torsional and deforming forces acting on the distal humerus [42].

To achieve the necessary compression, stability, and rigid fixation, a strategy based on four key principles was employed; these involved parallel plating, supracondylar shortening, iliac crest autograft, and contracture release [5]. Among the studies reviewed, only 3 of the 14 did not utilize a bone graft during revision ORIF. The most common approach combined a rigid construct with biological support. While fracture stability was generally achieved using this method, elbow stiffness was also frequently observed, representing a significant postoperative challenge for surgeons. Across the included studies, a success rate of 90% was demonstrated for ORIF.

The review indicated a success rate of 74% for TEA; in comparison, ORIF achieved an even higher success rate of 90% when indications were appropriately applied. However, several studies noted that although nonunion cases treated with ORIF healed effectively, there was often limited postoperative ROM of the elbow, averaging between 80° and 90°, and several patients experienced concurrent nerve symptoms [5, 12, 43]. Overall, patients treated with ORIF were more likely to achieve union compared with other strategies examined.

Demographic data revealed that the ORIF group generally consisted of younger patients with a more balanced gender distribution compared with other treatment groups. These factors were considered to have influenced the outcomes, as better healing and functional recovery were typically experienced by younger patients. For very complex distal humerus nonunion cases, a two-stage approach was often advocated: initial surgery aimed at achieving union and restoring motion, followed by a second procedure to further improve movement once union was established [44, 45]. To facilitate strong compression and ensure proper contact between the proximal and distal fragments, metaphyseal shortening was sometimes employed. Shortening up to 2 cm (or 3 cm in severe cases) was generally well tolerated and did not significantly weaken the triceps [46].

In cases where the bone defect resulting from resection was too large to achieve compression through shortening alone, the use of structural autografts—typically harvested from the iliac crest—was recommended. Stable fixation was best achieved with two parallel plates and long interdigitating screws. Several authors preferred a 90°–90° configuration [17], particularly in cases involving coronal plane fractures [47]. In exceptional cases requiring enhanced stability, a third plate could be added in a buttress configuration.

Regarding functional outcomes, ORIF results were comparable to those of TEA, with approximately 15% of patients demonstrating fair function. Notably, ORIF produced marginally higher rates of excellent function, suggesting that, although healing rates were superior with ORIF, the overall functional outcomes were similar between the two methods[48].

### Infected nonunion

The primary surgical indication was infected cases of distal humerus nonunion. Various surgical interventions were performed: 1 case was managed with a two-stage reconstruction, consisting of bone and soft tissue debridement followed by ORIF with a vascularized bone graft; 23 cases were treated with Ilizarov external fixation alone; and 6 cases received Ilizarov external fixation combined with iliac crest harvesting (Table 4).

Functional outcomes were generally unavailable in most of the analyzed studies. When reported, the results were predominantly mediocre, with 44% achieving good functionality and 54% achieving fair functionality. Despite this, approximately 96% of patients demonstrated radiographic or clinical healing at the most recent follow-up.

## Study limitations

The review incorporated studies of diverse designs, including randomized controlled trials, cohort studies, and case reports, with varying methodologies. While data on different surgical techniques were presented, the number of studies reporting on less common procedures, such as the Ilizarov technique for infected nonunions, was limited. This limitation potentially restricted the generalizability of the findings concerning these techniques. The mean follow-up duration across studies varied considerably, which may have influenced the evaluation of long-term outcomes. Shorter follow-up periods could have led to an underestimation of complication or failure rates that might have emerged later.

## Conclusions

The ORIF revision approach demonstrated a higher success rate for the treatment of aseptic nonunion compared with other modalities, suggesting that it might be considered the preferred option for clinicians; however, individual patient factors needed to be taken into account. Surgical expertise and thorough preoperative assessment were recognized as crucial for achieving successful outcomes. Further research was recommended to elucidate the long-term results associated with different procedures.

## Supplementary Information

Supplementary Material 1.

## Data Availability

All data used for this systematic review of literature have been retrieved from PubMed.

## Abbreviations

ORIF: Open reduction and internal fixation
TEA: Total elbow arthroplasty
ROM: Range of motion
mCMS: Modified Coleman Methodology Score
PRISMA: Preferred Reporting Items for Systematic Reviews and Meta-analyses
DASH: Disabilities of the arm, shoulder, and hand
MEPS: Mayo Elbow Performance Score
EF: External fixator
TC: Computed tomography
RSA: Reverse shoulder arthroplasty
