# Supplementary material for: Extraarticular distal humeral nonunion: systematic review of literature
Source: J Orthop Traumatol. 2025 Jul 21;26:48. doi: 10.1186/s10195-025-00861-y (PMC12279640; doi:10.1186/s10195-025-00861-y)
Supplement: Supplementary file 1 — Supplementary Material 1. [file 10195_2025_861_MOESM1_ESM.docx]

**Supplementary**

**MESH** ( Medical Subject Headings)

(("Humeral Fractures"[MeSH] OR "Fractures, Ununited"[MeSH] OR "humerus fracture nonunion"[tiab] OR "distal humerus nonunion"[tiab] OR "pseudoarthrosis of the humerus"[tiab])

AND

("Fracture Fixation, Internal"[MeSH] OR "Open Reduction Internal Fixation"[tiab] OR "ORIF"[tiab] OR "Bone Transplantation"[MeSH] OR "bone graft"[tiab] OR "Ilizarov Technique"[MeSH] OR "Ilizarov"[tiab] OR "Arthroplasty, Replacement, Elbow"[MeSH] OR "Total Elbow Arthroplasty"[tiab] OR "TEA"[tiab])

AND

("Fracture Healing"[MeSH] OR "Treatment Outcome"[MeSH] OR "Range of Motion, Articular"[MeSH] OR "functional outcome"[tiab] OR "range of motion"[tiab] OR "complications"[tiab]))
